# Proprioceptive contribution to oculomotor control in humans

**DOI:** 10.1002/hbm.26080

**Published:** 2022-09-22

**Authors:** Daniela Balslev, Alexandra G. Mitchell, Patrick J. M. Faria, Lukasz Priba, Jennifer A. Macfarlane

**Affiliations:** ^1^ School of Psychology and Neuroscience University of St Andrews St Andrews UK; ^2^ Clinical Research Centre Ninewells Hospital Dundee UK; ^3^ Medical Physics Department NHS Tayside, Ninewells Hospital Dundee UK; ^4^ Present address: Center for Functionally Integrative Neuroscience Aarhus University Aarhus Denmark; ^5^ Present address: Woodgrange Infant School London UK; ^6^ Present address: NHS Education for Scotland Edinburgh UK

**Keywords:** extraocular muscles, eye, human, oculomotor, proprioceptive, visual

## Abstract

Stretch receptors in the extraocular muscles (EOMs) inform the central nervous system about the rotation of one's own eyes in the orbits. Whereas fine control of the skeletal muscles hinges critically on proprioceptive feedback, the role of proprioception in oculomotor control remains unclear. Human behavioural studies provide evidence for EOM proprioception in oculomotor control, however, behavioural and electrophysiological studies in the macaque do not. Unlike macaques, humans possess numerous muscle spindles in their EOMs. To find out whether the human oculomotor nuclei respond to proprioceptive feedback we used functional magnetic resonance imaging (fMRI). With their eyes closed, participants placed their right index finger on the eyelid at the outer corner of the right eye. When prompted by a sound, they pushed the eyeball gently and briefly towards the nose. Control conditions separated out motor and tactile task components. The stretch of the right lateral rectus muscle was associated with activation of the left oculomotor nucleus and subthreshold activation of the left abducens nucleus. Because these nuclei control the horizontal movements of the left eye, we hypothesized that proprioceptive stimulation of the right EOM triggered left eye movement. To test this, we followed up with an eye‐tracking experiment in complete darkness using the same behavioural task as in the fMRI study. The left eye moved actively in the direction of the passive displacement of the right eye, albeit with a smaller amplitude. Eye tracking corroborated neuroimaging findings to suggest a proprioceptive contribution to ocular alignment.

## 1 INTRODUCTION

Without precise control of the rotation of the eyes in the orbits, it would be difficult to engage in activities that require accurate vision. To estimate eye position, the central nervous system has access to several sources. One of them is the signal from proprioceptive receptors in the extraocular muscles (EOMs) known as oculoproprioceptive feedback (Balslev & Miall, 2008; Gauthier et al., 1990; Han & Lennerstrand, 1999; Knox & Whalley, 1997; Skavenski, 1972; Velay et al., 1994; Wang et al., 2007). The other sources are the corollary discharge (Sommer & Wurtz, 2002) and the visual feedback (Poletti et al., 2013).

A theoretical argument can be made for using all available information to optimize precision. For the hand for instance, combining sensory sources makes position information more robust (van Beers et al., 2002). Experimental support for the role of oculoproprioception in the feedback control of eye movements comes from behavioural studies where this input was reduced or altered (Weir et al., 2000). In humans, during sustained passive deviation of one eye, the velocity of smooth pursuit or the amplitude of saccades executed by the other eye decreases (Knox et al., 2000; Van Donkelaar et al., 1997). In contrast, experiments in macaques have raised doubts about the contribution of this sensory modality to feedback oculomotor control. For instance deafferentation studies in macaques show no immediate effect of the lack of oculoproprioceptive feedback on the accuracy of saccades, smooth pursuit or vergence (Guthrie et al., 1983; Lewis et al., 2001) and no decrease in the precision of the estimate of eye position (Lewis et al., 1998).

One possible explanation for the discrepancy between animal models and human data is the interspecies differences in the function of the oculoproprioceptive receptors. For instance, whereas human EOMs have numerous muscle spindles (Donaldson, 2000), this type of receptor is rare in macaques (Greene & Jampel, 1966; Maier et al., 1974).

The extraocular motor nuclei in the macaque do not respond to the proprioceptive inflow resulting from muscle stretch (Keller & Robinson, 1971). In humans, electromyographic studies have returned conflicting results about whether a monosynaptic stretch reflex in the EOMs exists (Breinin, 1957; Irvine & Ludvigh, 1936). It remains unknown whether the proprioceptive input from the EOMs has any effect on the activity of the human extraocular motor nuclei.

Recent progress in functional magnetic resonance imaging (fMRI) data acquisition and data analysis allows to visualize the function of relatively small structures of the brainstem such as the abducens or oculomotor nuclei in humans (Furlan et al., 2015; Linzenbold et al., 2011). Here, we used these methods to find out whether the extraocular motor nuclei respond to oculoproprioceptive input.

To isolate oculoproprioceptive feedback from an associated oculomotor command we used passive eye movement. Examining the proprioceptive input from the EOMs is more challenging than that of the skeletal muscles because the eye is more vulnerable to mechanical trauma. Three approaches have been used in humans. Firstly, one can passively rotate the eyeball. One can do so in a controlled way using a scleral lens attached to the cornea by suction to fix the eye in a displaced position (Balslev et al., 2012; Gauthier et al., 1990; Gauthier et al., 1994; Knox et al., 2000). Because applying and removing the lens takes minutes, such a task cannot separate the immediate effect of proprioceptive stimulation from adaptive changes in the oculomotor system in response to abnormal proprioception. An alternative that overcomes this problem was proposed by Bridgeman and colleagues (Bridgeman & Delgado, 1984; Bridgeman & Stark, 1991; Ilg et al., 1989). The participants place their own index finger on the eyelid, at the outer canthus, to briefly (<1 s) push on the eyeball. The eye press is brief, painless and can easily be adapted for the fMRI scanning environment (Balslev et al., 2011). This is the method we used in this study. This method however has some disadvantages. The strength and the timing of the push cannot be fully controlled. Furthermore the finger movement itself causes brain activation and tactile stimulation of the eyelid. To address these concerns, we instructed the participants to exert the minimum force that passively moved the eyeball (see Section 2) and added an auditory prompt for the onset of the finger press. We also included control conditions to rule out brain activation caused by finger movement or tactile stimulation on the eyelid. Secondly, one could examine EOM proprioception using high‐frequency/low‐amplitude EOM tendon vibration via an electromagnetic vibrator applied on the eyelid. Vibration can trigger the illusory perception of eye movement in the absence of an actual movement (Velay et al., 1997). The authors note, however, that when the eye did not move, the illusion was weaker and variable across trials (Velay et al., 1997). Finally, inhibitory repetitive transcranial magnetic stimulation over the anterior parietal cortex in humans is the most recent method devised to reduce the processing of EOM proprioceptive inflow in the somatosensory cortex (Balslev & Miall, 2008; Odoj & Balslev, 2013). The brainstem, however, is currently outside the reach of this method because of the attenuation of the electric field with the distance from the electromagnetic coil.

Assessing the effect of EOM proprioception on the activity of the extraocular motor nuclei using the eye press task requires the absence of visual stimuli. In the presence of visual stimuli, the retinal slip caused by passive eye movement prompts compensatory EOM contraction. This contraction is not necessarily related to the proprioceptive intervention. A previous eye tracking experiment showed that when visual targets are present, the pressed eye rotates much less compared with a condition when visual targets are absent (Ilg et al., 1989). Because complete darkness is difficult to achieve in the clinical MRI scanner, the participants were scanned with their eyes closed. This precluded eye tracking during the fMRI experiment.

Because the fMRI results identified a proprioceptive projection from the right eye to the extraocular motor nuclei that control the horizontal movement of the left eye, a follow‐up eye tracking experiment sought to corroborate these findings by examining the movement of the left eye during the passive rotation of the right eye in complete darkness. Previous eye tracking using the same task (Ilg et al., 1989) found small fixational movements of the left eye around a resting baseline during the right eye finger press. No statistical analysis of the eye traces was conducted in that study. Visual inspection of the eye traces suggested that in at least some trials, the active movements of the left eye mirrored the passive movements of the right eye, albeit with a smaller amplitude. We examined therefore the hypothesis that the passive rotation of the right eye was associated with active movement of the left eye in the same direction as the passive displacement.

## 2 METHODS

### 2.1 Participants

Healthy, right‐handed adults participated after giving written informed consent. Sixteen participants (12 women, 4 men, median age 25, range 19–38) were recruited for the fMRI experiment and 17 participants (14 women, 3 men, median age 22, range 19–46) were recruited for the follow‐up eye tracking experiment. Handedness was assessed by self‐report. The participants were asked one question about which hand they preferred to use for skilled activities like writing. A single‐item assessment of handedness shows high classification concordance with more extended inventories (Coren, 1993). Data from two participants in Experiment 2 were excluded because of blinks or saccades. The study was approved by the Ethics Committee of the School of Psychology and Neuroscience at the University of St Andrews (PS11859) and the NHS Research Ethics Service (14/NW/1525). No statistical methods were used to predetermine sample size. The number of participants was chosen based on previous fMRI studies that used similar methods (Balslev et al., 2011; Furlan et al., 2015; Himmelbach et al., 2013; Linzenbold et al., 2011).

### 2.2 fMRI experiment

#### 2.2.1 Task

Before the experiment, participants practiced pressing their right eye gently while viewing an object through both eyes and increased the force gradually until they experienced double vision. An eye press of a strength that produces double vision during normal binocular viewing was assumed to be sufficient to passively displace the eyeball in complete darkness. The reason why the displacement is much smaller in normal light conditions is thought to be the retinal slip, which triggers an EOM contraction that opposes the push (Ilg et al., 1989). We verified that the pushed eye moved, while the participants tried the task in normal light conditions, with both eyes open. A follow‐up eye tracking experiment in complete darkness (see Section 2.3) confirmed that the finger press reliably rotated the eyeball by ~12°.

The participants wore headphones inside the MRI head coil. Their right hand rested on their cheek inside the coil, so that the index finger could easily reach the outer canthus of the right eyelid. Head movement was restrained using soft pads.

The participants kept their eyes closed throughout the scanning session. There were four different behavioural conditions: (a) *passive* (when prompted by a tone, the participant briefly pushed the right eye medially with their right index finger, which touched the eyelid at the outer canthus), (b) *touch* (when prompted, the participant touched the eyelid at the same location, without moving the eyeball), (c) *active* (when prompted, the participant shifted their gaze, with eyes closed, to one side then back to the central position) and (d) *rest* (listening to sounds with the eyes closed).

Trials of each condition were grouped in 25 s blocks. Each block began with a verbal instruction (4.8 s). After the instruction, a series of tones cued the start of each trial. The duration of the tone was 100 ms. The inter‐tone interval was chosen randomly from a normal distribution with a mean of 2 s and a standard deviation of 0.5 s (range 1.34–2.82 s). The median number of trials in each block was 10 (range 9–11). Tones were played in the same way in all blocks. The participants performed each condition block four times within each 400‐s run. Each participant completed ~10 runs (range 6–11, median 10). Block order was counterbalanced across runs and participants. Stimuli were generated in Psychophysics Toolbox v. 3 (Brainard, 1997).

#### 2.2.2 Data acquisition

We used a 3 Tesla MRI scanner (Siemens PrismaFIT™, Erlangen, Germany) with a 20‐channel head and neck coil. Twelve runs were acquired. Each run consisted of 133 T2* weighted gradient echo EPI volumes (slice thickness 2.0 mm, 25 slices interleaved acquisition, TR = 3090 ms, TE = 44 ms, FOV 128 × 128 mm, matrix 64 × 64). Participants were given a short break after the first six runs. We used a small field‐of‐view acquisition protocol (ZOOMit). EPI slices were oriented coronally, parallel with the midline of the brainstem at the level of the pons, to cover the entire brainstem. This small field of view allowed us to visualize the extraocular motor nuclei of the brainstem with adequate spatial resolution, but excluded most of the cerebral cortex and the cerebellum.

For anatomical localisation, high‐resolution T1‐weighted structural images were acquired in sagittal direction using an MP‐RAGE sequence (slice thickness = 1 mm, TR = 1900 ms, TE = 2.64 ms, FOV 200 × 200 mm, matrix 256 × 246). One whole‐brain EPI image was acquired in each participant by increasing the number of slices. This was used to co‐register the small field‐of‐view EPI images to the structural image.

#### 2.2.3 Statistical analysis

Data was analysed with SPM12 (Friston et al., 1995). The images were slice‐timing corrected, realigned and unwarped, spatially normalized to MNI152‐template (ICBM) using the Tissue Probability Map by (Lorio et al., 2016) and then smoothed with a 3 mm FWHM filter. The alignment of the brainstem EPI images with the structural scans (Figure S1) and with the MNI152‐template (Figure S2) was verified by visual inspection.

The design matrix for single‐subject analyses included four regressors (*passive*, *touch*, *active* and *rest*). Event timing was calculated from the vector of onset for the pacing tone by adding 0.4 s to approximate the participant's reaction time. All events were modelled by convolving the event onset vectors with the hemodynamic response function. To account for head motion, the six parameters from the realignment transformations (three translations, three rotations) were added to the design matrix. Runs with more than 2 mm of head motion were discarded (13 runs in total from four participants). The cut‐off frequency for high‐pass filtering was 1/128 s.

To identify areas that receive EOM proprioceptive input we used a random effects analysis with the conjunction (*passive–rest*) AND (*active–rest*) masked exclusively with the contrast (*touch–rest)*. The exclusive mask had a liberal threshold (voxel‐level *p* < .05, uncorrected for multiple comparisons) to remove all voxels that showed any evidence of increased activity during tactile stimulation of the eyelid or finger movement. The null hypothesis for the conjunction analysis was [NOT (*passive–rest*)] OR [NOT (*active–rest*)] (Friston & Penny, 2005). The threshold for statistical significance was voxel‐level *p* < .05, corrected for multiple comparisons using family‐wise error. The correction for multiple comparisons was done within predefined regions of interest (ROI). This is a common solution to the low signal‐to‐noise ratio problem in brainstem fMRI.

#### 2.2.4 Regions of interest

Because the passive condition stretched the lateral rectus muscle of the right eye and the active condition instructed a horizontal saccade, potential activation was expected in the oculomotor and abducens nuclei, which innervate the medial and lateral rectus muscles, respectively (Horn & Leigh, 2011). In addition, the superior colliculus was designated as a ROI. This is because the superior colliculus receives input from the spinal trigeminal nucleus (Harting et al., 1997) and because eye position modulates the activity of neurons within this structure (Campos et al., 2006; Mullette‐Gillman et al., 2009; Van Opstal et al., 1995).

There is currently no probabilistic brainstem atlas, though efforts are underway (García‐Gomar et al., 2019). To define the ROIs, we used the peak activation coordinates for the superior colliculi (Furlan et al., 2015; Linzenbold et al., 2011), oculomotor nuclei (Linzenbold et al., 2011) and abducens nuclei (Beissner, 2015; Linzenbold et al., 2011) from previously published studies that examined voluntary saccades. When more than one coordinate within the same structure were available, the centre of the ROI was calculated as their average. Table 1 shows the coordinates for the centre of the ROIs. The radius of the spherical ROIs was 4 mm, which is twice the standard deviation of the coordinates of the superior colliculi across participants (Furlan et al., 2015). Anatomical location was verified using the Duvernoy atlas (Duvernoy, 2004).

**TABLE 1:** Brainstem oculomotor regions active during proprioceptive stimulation of the right eye's lateral rectus muscle

Reference coordinates for ROI centre				Activity peaks within ROI				
	*x*	*y*	*z*	*x*	*y*	*z*	*Z*‐score	FWE‐corrected *p*‐value
Oculomotor nucleus								
L/R	0	−26	−11	−2	−24	−8	3.46	.02
Abducens nucleus								
L	−2	−41	−38	−4	−38	−38	2.96	.05
R	6	−42	−37		None			
Superior colliculus								
L	−4	−31	−4	−2	−28	−2	3.47	.02
R	5	−30	−4		None			

*Note*: The table shows activity peaks above the threshold of *p* < .05 corrected for multiple comparisons using family‐wise error (FWE) within the regions of interest. The analysis (*passive–rest*) AND (*active–rest*) masked exclusively with (*touch–rest)* (see Methods) was conducted within spherical regions of interest (ROI) centred on reference coordinates for the superior colliculi, oculomotor and abducens nuclei. The reference coordinates for each ROI centre are averages across coordinates reported in previous fMRI studies of saccadic eye movements (Beissner, 2015; Furlan et al., 2015; Linzenbold et al., 2011).

Although an oculoproprioceptive projection to the spinal trigeminal nucleus is likely (Manni et al., 1971; Porter, 1986), our analysis would not have been sensitive to detecting an oculoproprioceptive projection there. This is because this structure also receives tactile input from the eyelid area (Usunoff et al., 1997), and would therefore be excluded by the contrast (*touch–rest)*.

### 2.3 Eye‐tracking experiment

#### 2.3.1 Task

The behavioural task was similar with that of the *passive* condition of the fMRI experiment, with one notable difference. The participants kept their eyes open to allow eye tracking. The experiment was conducted in complete darkness to remove any visual contribution to the stability of gaze. The participants sat with the head fixed in a chin rest and cheek pads in front of an OLED monitor (Sony, Trimaster) with no backlight. The monitor was placed at 60 cm from their eyes. Each trial started with the onset of a central fixation target (the symbol “+”) in red on a black background, subtending 1° visual angle, on for 500 ms. One second after the fixation cross was extinguished a pre‐recorded audio instruction prompted the participants to press the left eye. Two seconds later an on‐screen instruction prompted them to blink. After another 2.5 s, a new trial started. The participants were asked to keep their gaze at the location of the fixation cross even after the cross had disappeared, and to blink, if possible, only when instructed. Three trials were blocked into one session. Each participant completed four sessions (12 trials/participant). Psychophysics Toolbox v. 3 was used to generate stimuli and control the eye‐tracker.

#### 2.3.2 Eye tracking

The participants wore a head‐mounted, binocular, infrared eye‐tracker (Eyelink II, SR Research) that recorded pupil location at 250 Hz with a spatial resolution of 0.01°. The eye tracker was calibrated before each session, so horizontal pupil position could be measured as the deviation from a central fixation point in degrees visual angle. Both the right and the left pupils were tracked individually, at the same time.

#### 2.3.3 Statistical analysis

To find out whether the movement of the left eye mirrored the passive displacement of the right eye during this task, we calculated the net movement of the left eye during the leftward (push) and the rightward (rebound) phase of passive right eye displacement (Figure 2). Data were analysed in Matlab (MathWorks). For each trial the position of the right eye over time was visualized. One of the authors (Patrick J. M. Faria) identified by visual inspection of these time‐series the onset of leftward movement, the maximum amplitude of this movement and the end of the rebound (rightward) movement of the right eye (Figure 2a). We defined the following time intervals: “During Push” (right eye displacement leftwards) started from the onset of the right eye push and ended when the maximum amplitude of this displacement was reached. “During Rebound” (right eye movement rightwards) started from the maximum amplitude of the right eye displacement and ended at the onset of the resting baseline after the push (Figure 2a). Trials with blinks, saccades or a second push were excluded (7% of the trials). The net movement of the left eye was defined as the signed difference in left eye position between the start and end of each phase. The predictions were for a net movement of the left eye leftwards (negative) “During Push” and rightwards (positive) “During Rebound.” Linear trends in left eye position over time were removed ahead of this analysis to avoid the confounding effect of instrument drift. After verifying the assumption of normality, two‐tailed, single‐sample *t*‐tests compared the net movement of the left eye in each of these phases with zero.

## 3 RESULTS

### 3.1 fMRI experiment

#### 3.1.1 Statistically significant activation of the left oculomotor nucleus and left superior colliculus and subthreshold activation of the left abducens nucleus

**FIGURE 1:**
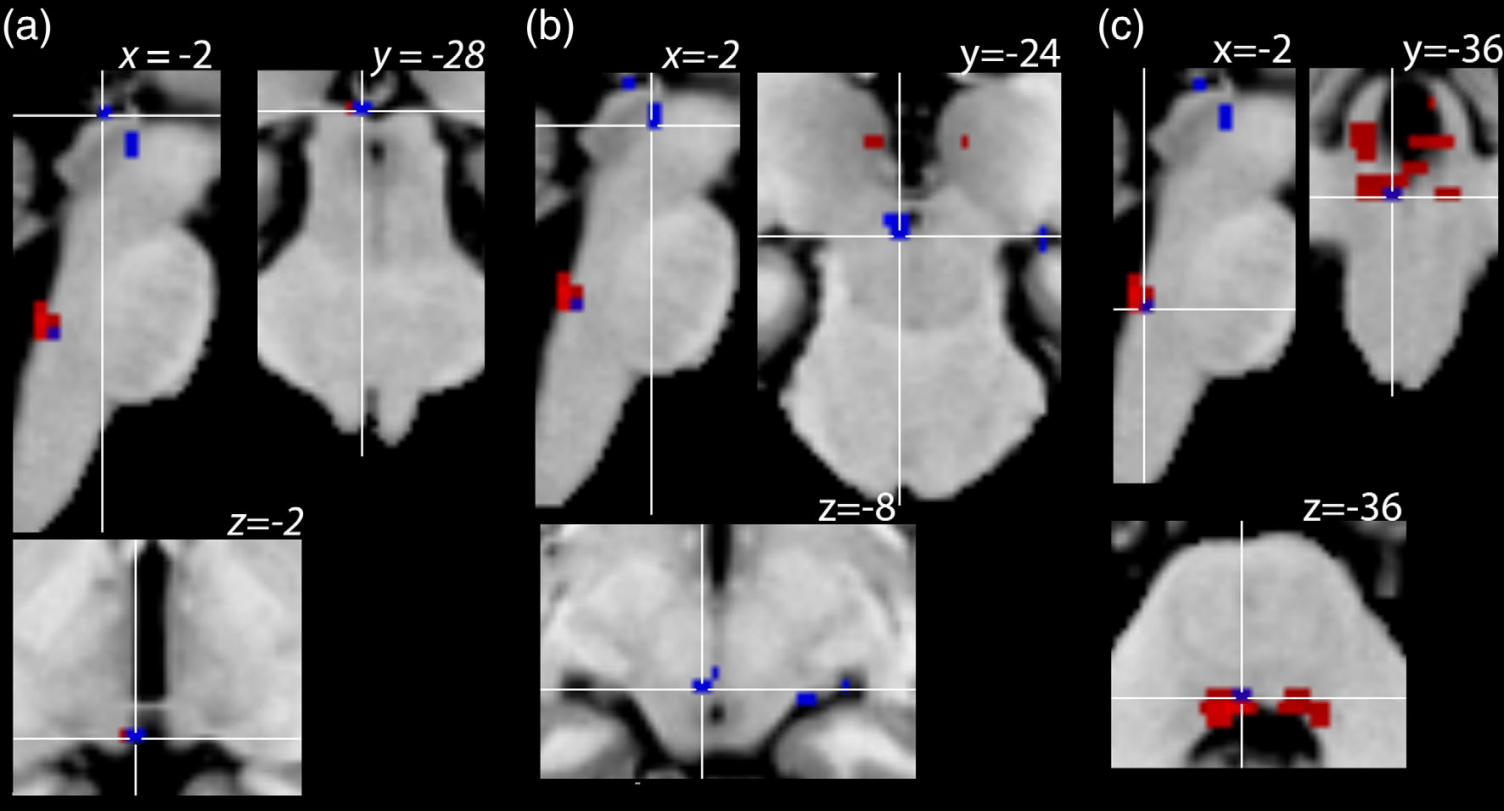
Brainstem areas where the neural activity increased in response to proprioceptive stimuli. Statistical parametric map for the conjunction (passive–rest) AND (active–rest) masked exclusively with (touch–rest) is visualised for the whole brainstem above the voxel‐level threshold *p* < .001, uncorrected for multiple comparisons (blue). To illustrate the specificity of these results for the left side of the brainstem, the threshold for visualisation here was lower than the threshold for statistical significance for the activation peaks listed in Table 1 (*p* < .05 corrected for multiple comparisons). Areas that responded to both active eye movements and passive EOMs stretch (blue) are overlaid on those that were significantly activated by active eye movements alone (red, visualised for the whole brainstem above the threshold of *p* < .05 corrected for multiple comparisons using family‐wise error for the entire brainstem). For anatomical localisation, statistical parametric maps are overlaid on the MNI152 (ICBM) template. Activity peaks are shown in three orthogonal projections: Sagittal (top left), coronal (top right) and transversal (bottom) at the level of the (a) superior colliculus, (b) oculomotor nucleus and (c) abducens nucleus. The crosshair in each panel indicates these nuclei

The left oculomotor nucleus, the left abducens nucleus and the left superior colliculus were active for both voluntary horizontal saccades in darkness and for a leftward press on the closed right eye (*p* < .05, *p* = .05, and *p* < .05, respectively corrected for multiple comparisons within the ROIs, Figure 1, and Table 1).

### 3.2 Eye‐tracking experiment

#### 3.2.1 Net active movement of the left eye in the same direction as the passive displacement of the right eye

None of the participants indicated any discomfort during the eye push. The right eye was displaced leftwards by −11.7° ± 3.5° (mean ± standard deviation). The maximum amplitude was reached after 0.39 ± 0.14 s. During this phase of the right eye rotation, there was a smaller, net movement of the left eye leftwards, in the same direction as the right eye (mean ± standard deviation, −0.18° ± 0.26°, one‐sample *t*‐test, *p* = .015). After the leftward rotation the right eye returned to a central, resting baseline. The movement of the right eye during this rebound phase had an amplitude of 11.7° ± 3.25° and a duration of 0.34 ± 0.14 s. The left eye too showed a directional reversal of its net movement during this phase (mean ± standard deviation, 0.20° ± 0.30°, one‐sample *t*‐test, *p* = .023).

## 4 DISCUSSION

The fMRI study found a statistically significant activation in the left oculomotor nucleus and subthreshold activity in the left abducens nucleus in response to stretch of the right lateral rectus muscle. Because these structures consist of extraocular motor neurons and internuclear neurons that target the extraocular motor neurons, these neuroimaging findings suggest a role for proprioception in oculomotor control. Complementing the fMRI results, eye traces in complete darkness showed a net active movement of the left eye in the same direction as the passive rotation of the right eye. These behavioural results corroborate fMRI findings and further suggest a role for proprioceptive feedback in ocular alignment.

### 4.1 The activity of the brainstem oculomotor nuclei is unlikely to be explained by finger movement, by the tactile stimulation on the eyelid or by the preparation of an eye movement

The task conditions in the fMRI study differed by the presence of finger movement, as well as by the tactile stimulation of the eyelid. Furthermore, preparing a finger movement could have prepared the oculomotor system to resist or to help the passive displacement. Although these confounds cannot be completely ruled out by the task design, we argue below that they are unlikely to explain the current findings.

Without the exclusive mask (*touch–rest*), the conjunction analysis (*passive–rest*) AND (*active–rest*) would identify not only brainstem regions that respond to both passive and active eye movement, but also those that respond to both finger and eye movement. One example of a brainstem region that is sensitive to both finger and eye movement is the superior colliculus, which is thought to orient towards or away from a stimulus across all body effectors (Gandhi & Katnani, 2011). To address this possible confound, the exclusive mask (*touch*–*rest*) removed any voxels for which there was statistical evidence of a response to the preparation or the execution of a finger movement. The threshold for this exclusive mask (*p* < .05 uncorrected for multiple comparisons, *Z* > 1.96) was more liberal than that of the conjunction analysis (*p* < .05 corrected for multiple comparisons, *Z* > 2.96). Even so, because the amplitude and strength of finger movement were likely to be higher in the *passive* than *touch* condition, one could argue that the activity in a brainstem area related to the preparation/execution of a finger movement could fail to reach the lower threshold of the comparison (*touch*–*rest*), while still reaching the higher threshold of the comparison (*passive–rest*). This would be the case if the BOLD response in that area scales with the force or the amplitude of finger movement. Such a pattern of activity has been described for instance in the primary motor cortex (Cheney & Fetz, 1980). To the best of our knowledge, however, neural activity that scales with the force or amplitude of finger movement has not been observed in the oculomotor or abducens nuclei, which are not involved in hand movements. These nuclei consist of motoneurons that innervate the EOMs and internuclear neurons that target such motoneurons (Horn & Leigh, 2011). It is unlikely that the increase in the BOLD signal in these nuclei in the passive condition reflects the preparation or execution of finger movement.

Likewise, because the conjunction analysis (*passive–rest*) AND (*active–rest*) was combined with the exclusive mask (*touch–rest*) whose threshold for statistical significance was lower than that of the conjunction, the tactile input on the eyelid is unlikely to explain the change in activity in the oculomotor or abducens nuclei.

Finally, preparation in the oculomotor system for a peripheral perturbation (for instance a contraction of the EOMs to withstand or to help the passive displacement) would be expected to affect the extraocular motor nuclei in the right brainstem. These nuclei innervate the right eye, which is the eye whose position was perturbed. Instead, fMRI results show a change in activity that was specific to the left motor nuclei. This specificity of the fMRI results for the left side of the brainstem (Table 1) was observed even at a lower threshold for statistical significance (*p* < .001, uncorrected for multiple comparisons, Figure 1).

### 4.2 The activation of the left oculomotor and abducens nuclei in response to oculoproprioceptive stimuli from the right lateral rectus muscle is not a monosynaptic stretch reflex

In the skeletal system, the proprioceptive inflow to the spinal motoneurons triggers a monosynaptic stretch reflex which maintains muscle contraction against external forces. In contrast, in the oculomotor system, where external perturbations are rare, the utility of such a reflex has been questioned. It was identified in rats and squirrel monkeys (Dancause et al., 2007), but not in cats (Tomlinson & Schwarz, 1977) or macaques (Keller & Robinson, 1971). The response in the left oculomotor or abducens nucleus in response to the stretch of the right, lateral rectus muscle observed here does not have the signature of a monosynaptic stretch reflex. This is because such a reflex would be expected to cause primarily the contraction of the stretched muscle itself, the right lateral rectus, and the activation of the ipsilateral right abducens nucleus, which innervates this muscle (Horn & Leigh, 2011; Miller et al., 2002; Müri et al., 1996). Instead, we found contralateral activation of the oculomotor nucleus; and contralateral, rather than ipsilateral, sub‐threshold activation in the abducens nucleus (Figure 1). This pattern of activity makes a monosynaptic stretch reflex an unlikely explanation of these findings.

The increase in the BOLD response in the extraocular motor nuclei that do not innervate the stretched EOM echoes previous electrophysiological observations in cats. Electrical activity in the cat oculomotor nucleus was recorded only in response to passive stretch of the EOMs that were not innervated by the nucleus being recorded from (Tomlinson & Schwarz, 1977). The latency of the response they observed was 0.03–0.17 s, indicative of a polysynaptic pathway (Tomlinson & Schwarz, 1977).

There is an apparent discrepancy between these neuroimaging results in humans, where an increase in the BOLD signal was recorded in the extraocular motor nuclei in response to passive muscle stretch and the electrophysiological findings in the macaque, which show no such response (Keller & Robinson, 1971). The BOLD signal is a measure of neural activity. The increase in the BOLD contrast in a spatially localised region of the brain directly and monotonically reflects the increase in neural activity (Logothetis et al., 2001). The increase in BOLD signal correlates more strongly with the increase in the local field potentials (which reflects synaptic activity and energy consumption) than with the multiunit activity (which reflects the frequency of action potentials) (Logothetis et al., 2001). One explanation of the discrepancy could be the difference in the signals measured by the two methods. Another explanation could be that Keller and Robinson recorded from the extraocular motor nuclei that innervate the stretched muscle only, whereas our results show that the increased BOLD signal occurred in the contralateral motor nuclei, that innervate the other eye. An increase in the neural activity of the contralateral extraocular motor nuclei would have been overlooked by their experiment.

### 4.3 Proprioceptive coupling between the two eyes

**FIGURE 2:**
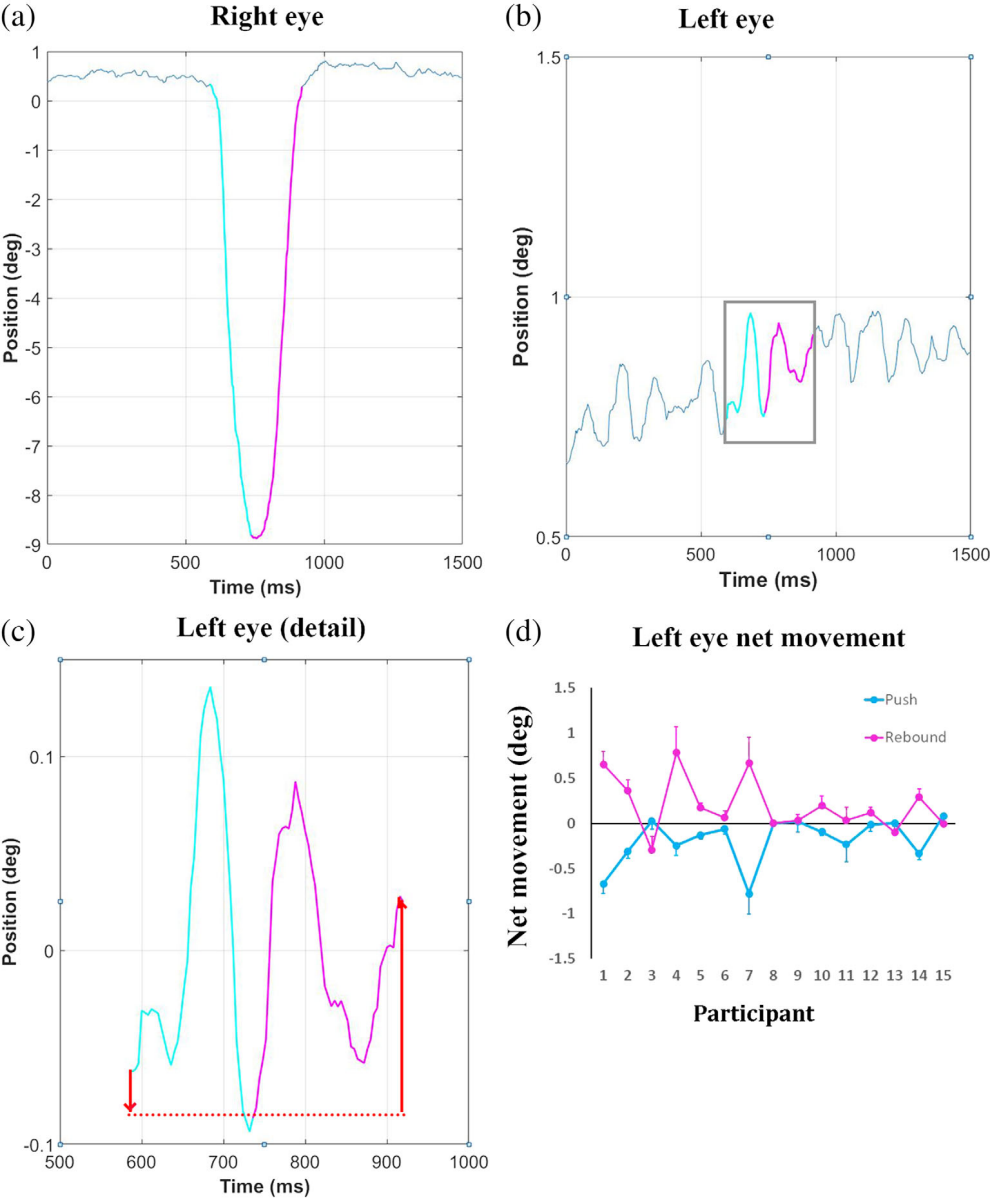
Eye movement in darkness during a brief push of the right eye leftwards. The push caused a transient leftward movement of the right eye followed by a rebound. The net active movement of the left eye mirrored the passive movement of the right eye. (a) Eye trace from one trial (participant 9) illustrating the passive movement of the right eye (cyan: Push; magenta: Rebound). (b) Eye trace of the left eye during the same trial. The colours indicate the active movements of the left eye during the two phases of the right eye displacement. (c) Detail of the left eye trace after removing any linear trend in the data. The red arrows show the net movement of the left eye during the push (cyan) and the rebound (magenta) phases of the passive right eye displacement. (d) Group data showing the net active movement of the left eye in the two phases of the passive displacement of the right eye in each individual participant. The error bars show ±1 standard error of the mean across trials calculated for each participant. Further examples of individual eye traces from participants 1–8 and 10–15 are available as Figures S3–S16

Eye‐tracking in complete darkness confirmed the passive movement of the right eye leftwards. After the push, the right eye returned to its initial position; presumably due to the elasticity of the orbital tissue and fixational eye movements. Although the amplitude of the left eye movement was much smaller than the passive right eye displacement, its net direction was the same as that of the right eye. This occurred during both the push and the rebound phase (Figure 2). This observation suggests a proprioceptive coupling between the two eyes. The absence of a match between the movement amplitude of the two eyes could reflect the smaller weight that proprioception has in the eye position estimate compared with the corollary discharge (Bridgeman & Stark, 1991; Gauthier et al., 1990).

These behavioural results corroborate neuroimaging evidence. The movement of the left eye leftwards and then rightwards during the passive right eye displacement explains the activity in both the abducens and oculomotor nuclei on the left side, which control the movement of the left eye in two opposite directions: laterally (leftwards) and medially (rightwards). We suggest that the proprioceptive input from the right eye was relayed to the oculomotor nuclei that control the movement of the left eye, to facilitate gaze alignment.

This suggestion would be in line with previous behavioural research in humans (Gauthier et al., 1994; Knox et al., 2000; Tamura & Mitsui, 1986; Van Donkelaar et al., 1997). For instance, during sustained passive deviation of one eye, the velocity of smooth pursuit or the amplitude of saccades executed by the other eye decreases (Knox et al., 2000; Van Donkelaar et al., 1997). Our behavioural results complement these previous findings by showing that also at shorter time scales (<1 s), the extraocular motor nuclei that move one eye respond to oculoproprioceptive stimuli from the other eye.

Finally, we would like to speculate about the proprioceptive receptors involved in this response. One candidate are the muscle spindles, which are responsible for a monosynaptic stretch reflex in the skeletal muscles and which are abundant in the human EOMs (Donaldson, 2000), but rare in the macaque EOMs (Greene & Jampel, 1966; Maier et al., 1974). Their involvement in the oculomotor response would offer one explanation for the discrepancy between the behavioural results in humans (Gauthier et al., 1994; Knox et al., 2000; Tamura & Mitsui, 1986; Van Donkelaar et al., 1997) vs. macaques (Lewis et al., 2001). Another candidate would be the palisade endings, which are common to both species, but whose function as proprioceptive receptors has been questioned (Lienbacher et al., 2011; Zimmermann et al., 2013). Because the palisade endings are more numerous in the EOMs of front‐eyed than lateral‐eyed mammals (Blumer et al., 2016) and because they are more numerous (Blumer et al., 2016) and more specialised (Lienbacher et al., 2019) in the medial rectus as opposed to other EOMs, it has been suggested that the palisade endings play a role in vergence.

## 5 CONCLUSION

Neuroimaging results showed an increase of activity in the human oculomotor system during EOM stretch. The oculoproprioceptive feedback from one eye was associated with an increase in the BOLD signal of the extraocular motor nuclei that innervate the other eye. Follow‐up behavioural results supported this evidence. Because the active movement of the left eye was coupled with the passive movement of the right eye, it is likely that this proprioceptive feedback plays a role in ocular alignment.

Understanding the interplay between sensation and movement in the oculomotor system could provide a more complete account of the disease mechanisms in non‐paretic infantile strabismus, where abnormal EOM proprioception has long been hypothesized (Bui Quoc & Milleret, 2014; Corsi et al., 1990; Dengis et al., 1998; Domenici‐Lombardo et al., 1992; Lennerstrand et al., 1997; Schiavi, 2016).

## AUTHOR CONTRIBUTIONS

**Daniela Balslev:** conceptualisation, methodology, supervision, software, analysis, writing—original draft, writing—review and editing, data curation, project administration, funding acquisition; **Alexandra G. Mitchell:** investigation, preliminary analysis (fMRI), writing—review and editing, software, methodology; **Patrick J. M. Faria:** investigation, analysis (eye tracking), writing—review and editing, methodology; **Lukasz Priba:** methodology (co‐designed fMRI data acquisition protocol), writing—review and editing; **Jennifer A. Macfarlane:** methodology (designed fMRI data acquisition protocol), supervision (fMRI data collection), writing—review and editing.

## CONFLICT OF INTEREST

The authors declare no conflict of interest.

## Supporting information


**Appendix S1** Supplementary Information

## Data Availability

The anonymized research data and code underpinning this research can be accessed at https://doi.org/10.17630/9e107884-df5a-4de6-8327-ba809d1b2168 (Balslev, 2022).
