# Supplementary material for: Proprioceptive contribution to oculomotor control in humans
Source: Hum Brain Mapp. 2022 Sep 22;43(16):5081–90. doi: 10.1002/hbm.26080 (PMC9582377; doi:10.1002/hbm.26080)
Supplement: Supplementary file 1 — Appendix S1 Supplementary Information [file HBM-43-5081-s001.docx]

**Supporting Material**

1. **Supplementary Results**

*Adequate spatial normalisation to allow anatomical localisation based on previous fMRI studies*

Anatomical structures visible in transversal sections (the intercollicular region or the posterior median sulcus) were found to be in alignment in functional and structural images (Figure S1). Likewise, an inspection of these landmarks in each participant’s mean EPI image shows adequate image alignment across participants (Figure S2).


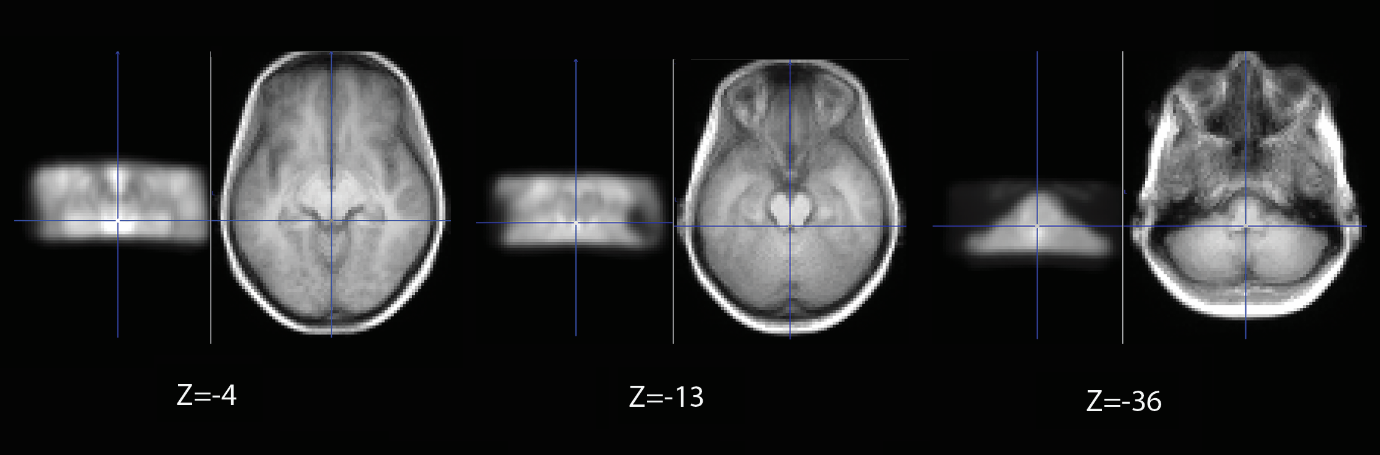


**Figure S1.** Transversal slices at three levels of the brainstem show co-registration of mean EPI (left) and mean anatomical T1 (right) scans across 16 participants. The crosshairs are yoked to show the same MNI coordinate in both functional and anatomical images. In both images they indicate the intercollicular region at (x, y, z) = (0, -35, -4) and posterior median sulcus at (0, -37, -13) and (0, -40*,* -36). Anatomical and functional scans are normalised to the MNI152 template (ICBM).


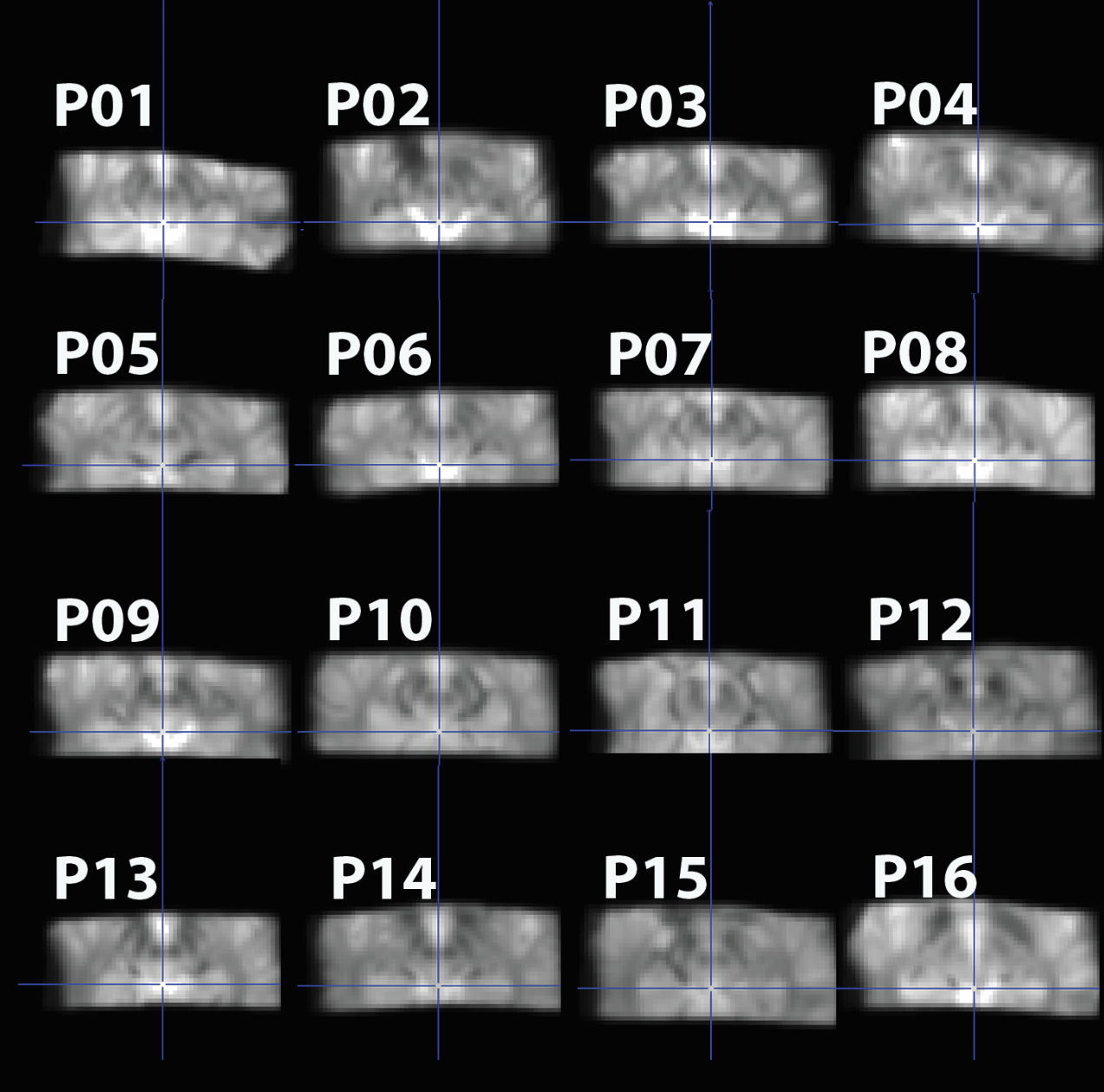
**Figure S2.** Transversal slices show the alignment between mean EPI images of individual participants (n=16) after co-registration with the MNI152 template (ICBM). The crosshair indicates the intercollicular region at the MNI coordinate (*x, y, z*) = (0, -35, -4) in all participants.


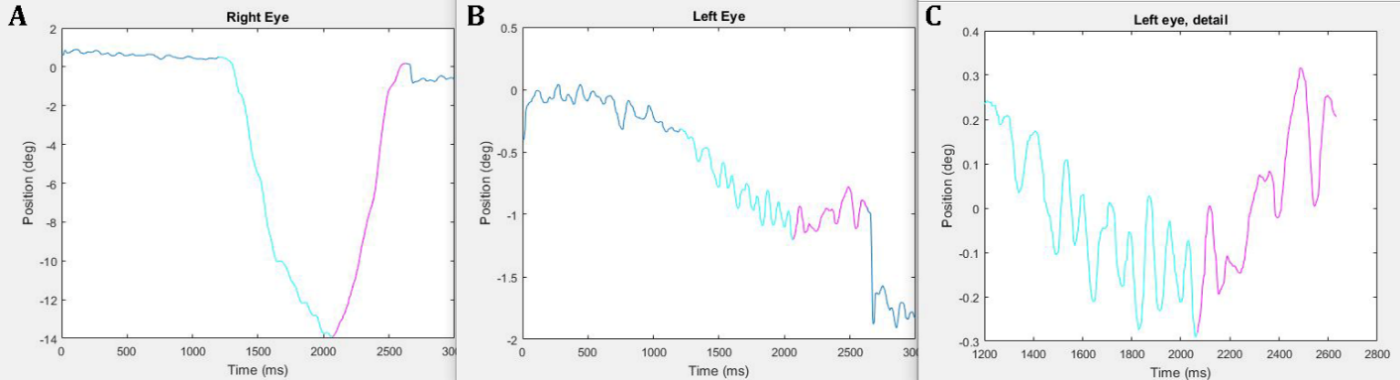


**Figure S3. Example eye trace in Participant 1.** A. Eye trace from one trial illustrating the passive movement of the right eye (cyan: push; magenta: rebound) **B**. Eye trace of the left eye during the same trial. The colours indicate the active movements of the left eye during the two phases of the right eye displacement **C.** Detail of the left eye trace after removing any linear trend in the data. The red arrows show the net movement of the left eye during the push (cyan) and the rebound (magenta) phases of the passive right eye displacement.


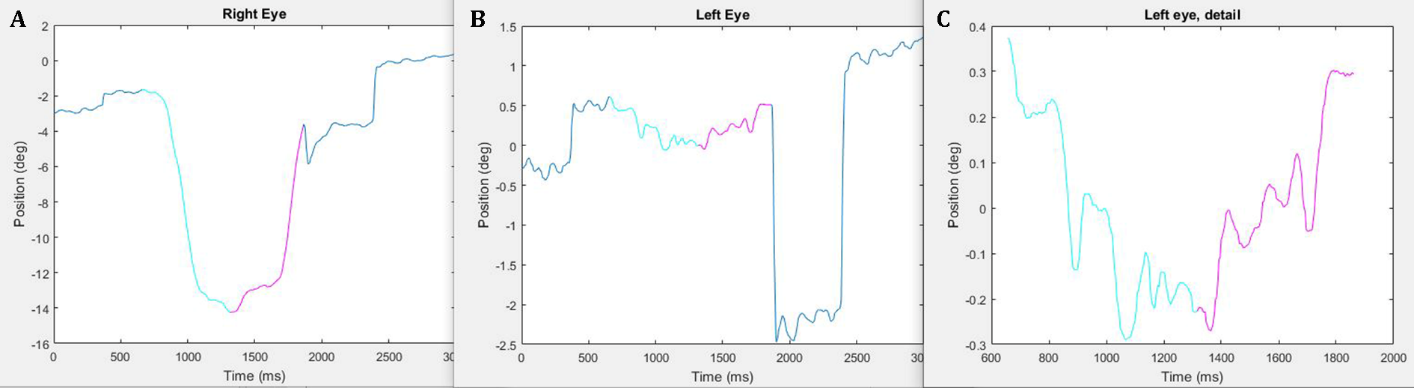
**Figure S4. Example eye trace in Participant 2.** Conventions like in Figure S3.


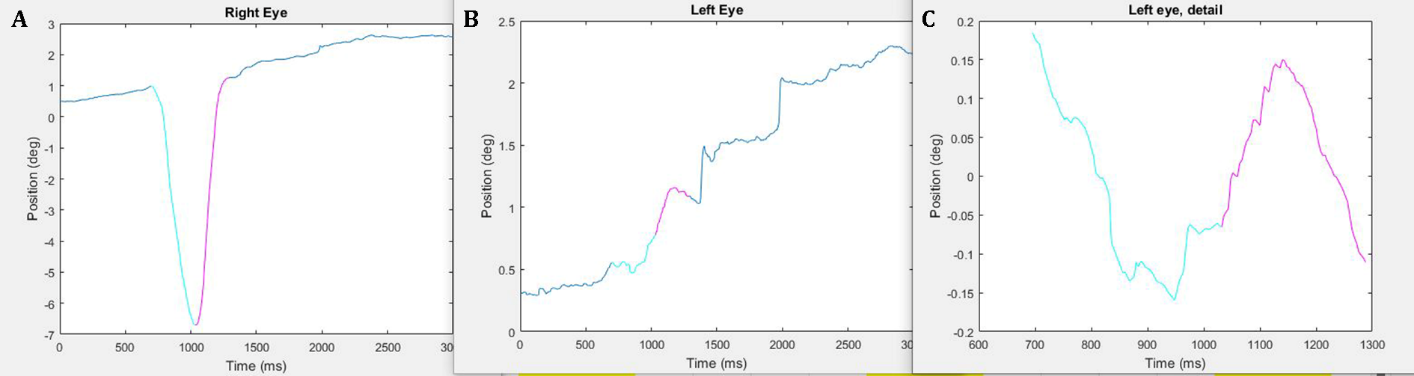
**Figure S5. Example eye trace in Participant 2.** Conventions like in Figure S3.


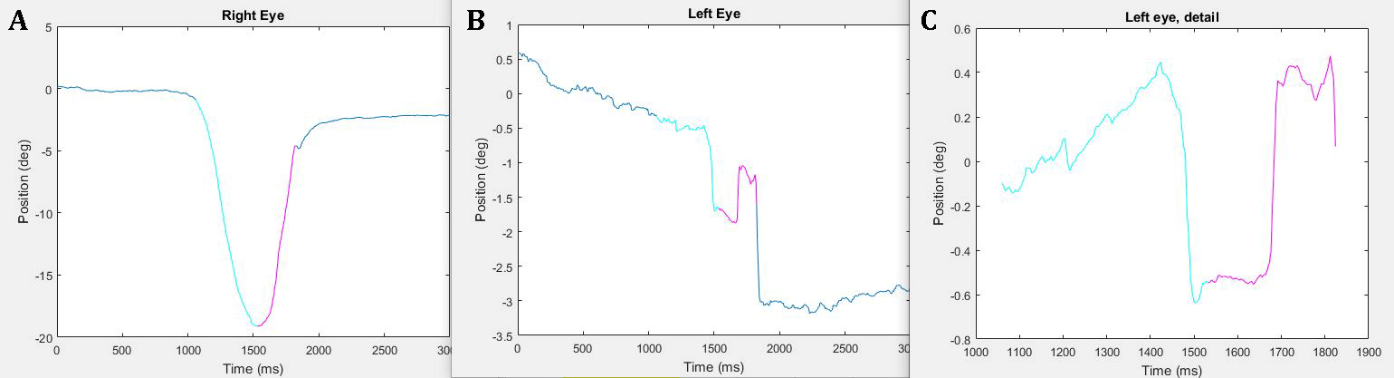
**Figure S6. Example eye trace in Participant 4.** Conventions like in Figure S3.


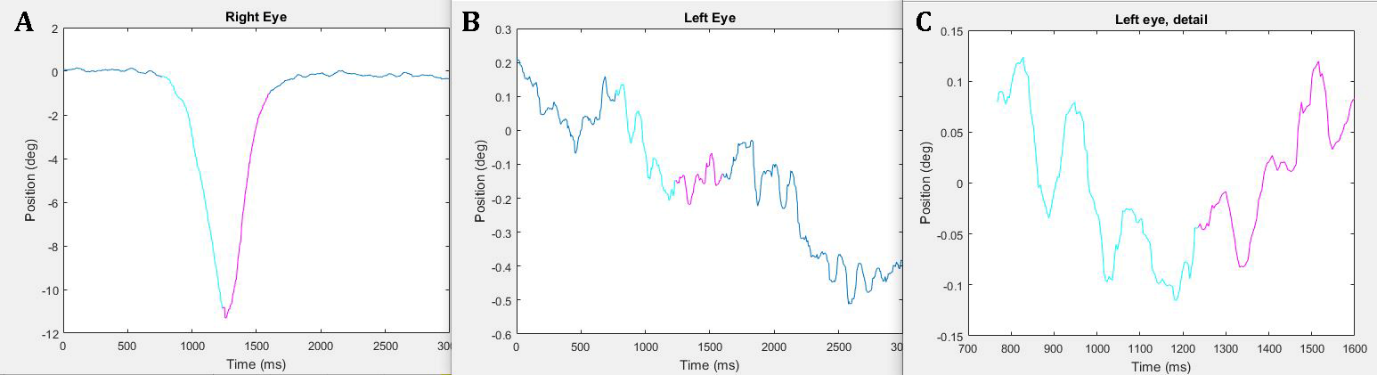
**Figure S7. Example eye trace in Participant 5.** Conventions like in Figure S3.


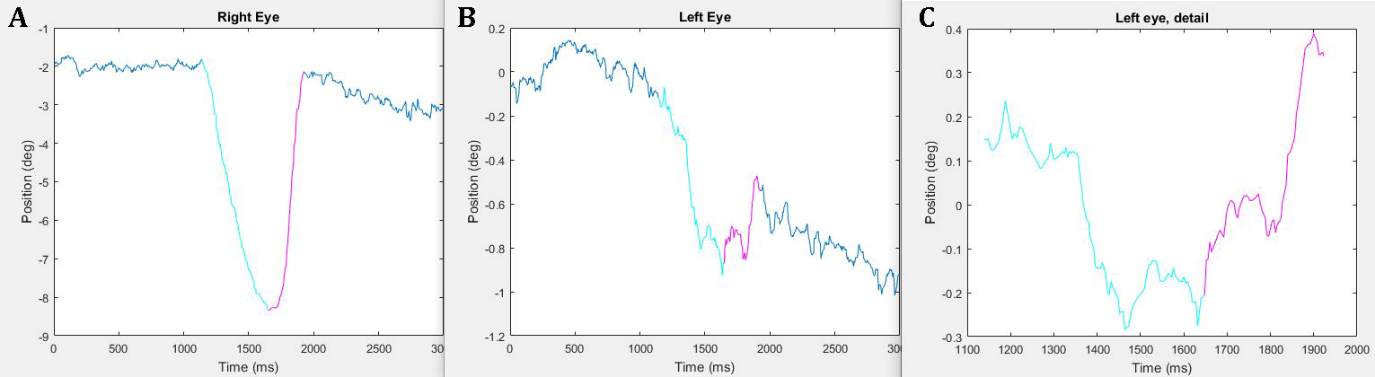


**Figure S8. Example eye trace in Participant 6.** Conventions like in Figure S3.


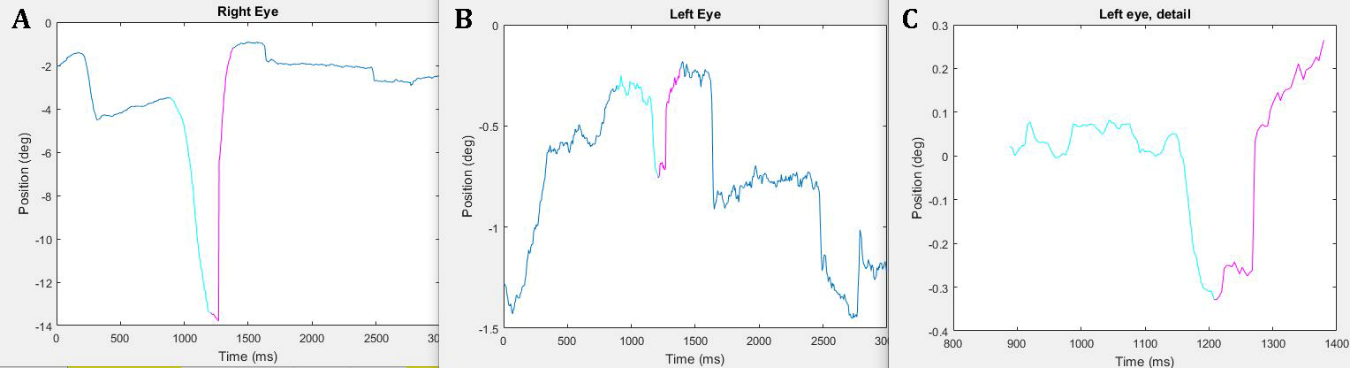


**Figure S9. Example eye trace in Participant 7.** Conventions like in Figure S3.


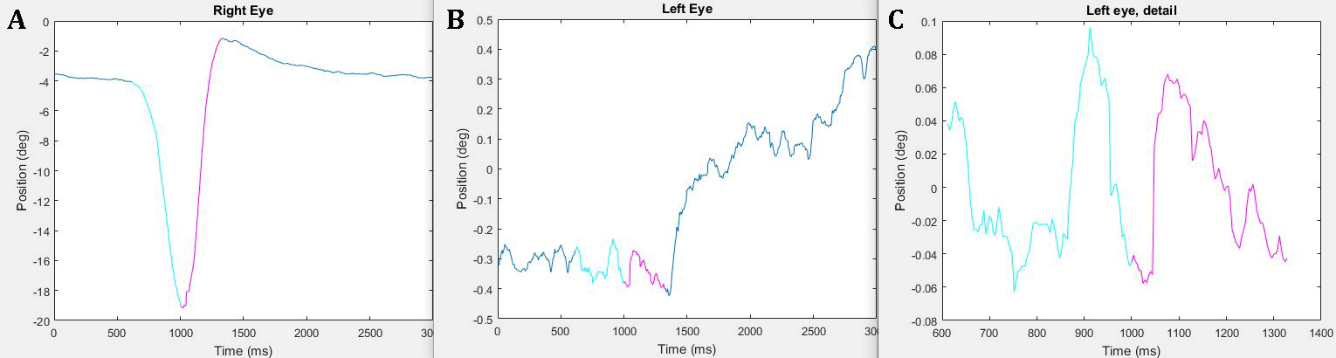
**Figure S10. Example eye trace in Participant 8.** Conventions like in Figure S3.

**Figure S11. Example eye trace in Participant 10.** Conventions like in Figure S3
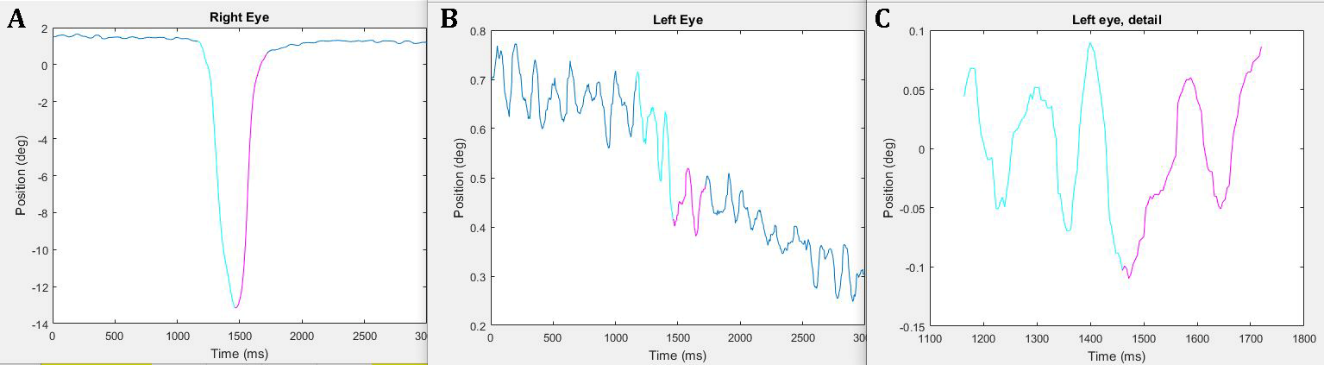


**Figure S12. Example eye trace in Participant 11.** Conventions like in Figure S
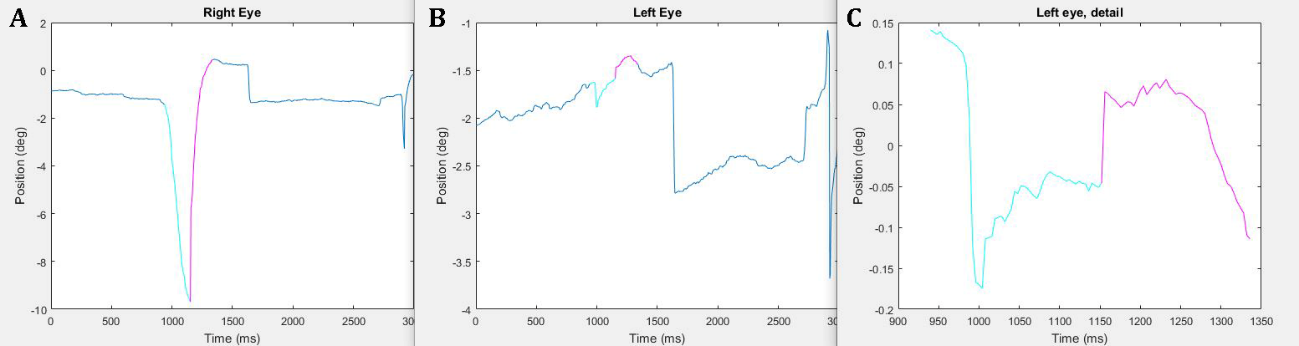
3


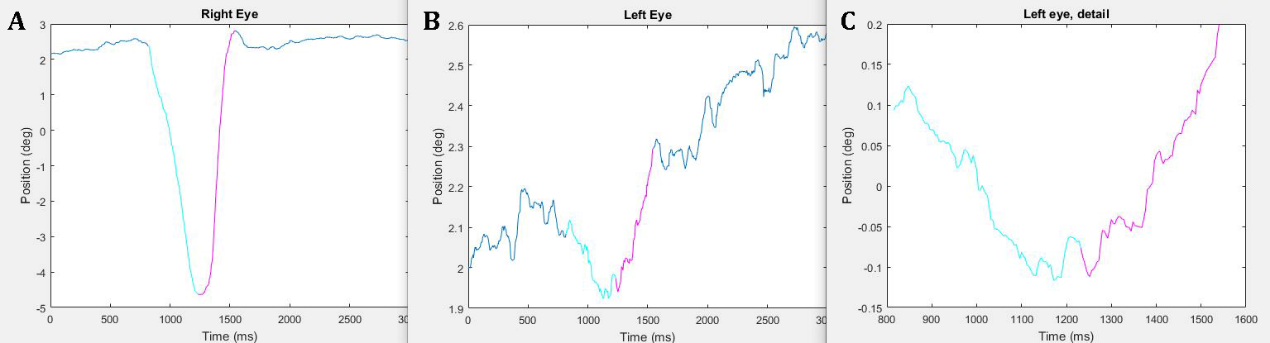
**Figure S13. Example eye trace in Participant 12.** Conventions like in Figure S3

**
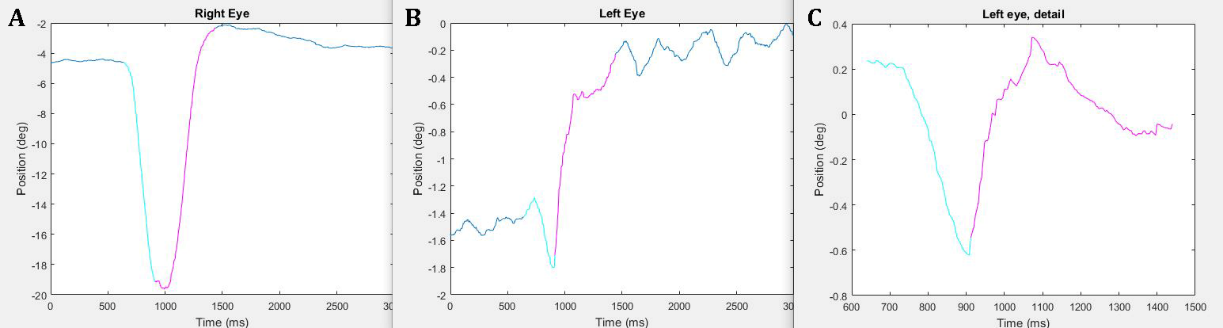
 Figure S14. Example eye trace in Participant 13.** Conventions like in Figure S3

**
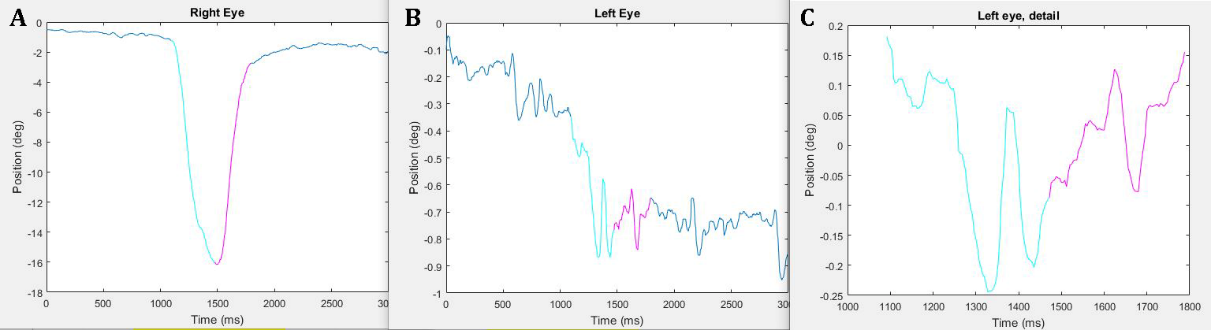
 Figure S15. Example eye trace in Participant 14.** Conventions like in Figure S3


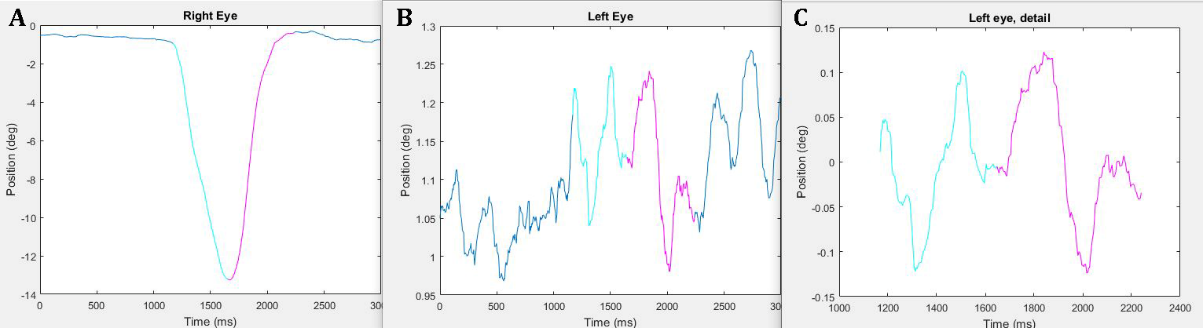
**Figure S16. Example eye trace in Participant 15.** Conventions like in Figure S3
